# Controlled mechanical properties and supramolecular chirality of hydrogels via pH change

**DOI:** 10.1016/j.mex.2019.02.017

**Published:** 2019-02-26

**Authors:** Ayesha Kousar, Chuanliang Feng

**Affiliations:** a State Key Lab of Metal Matrix Composites, School of Materials Science and Engineering, Shanghai Jiaotong University, Dongchuan Rd 800, 200240, Shanghai, China; b Henan Univ, Collaborat Innovat Ctr Nano Funct Mat & Applicat, Key Lab Special Funct Mat, Kaifeng 475004, China

**Keywords:** Method to improve mechanical properties and control chirality of supramolecular hydrogels, Hydrogel, Supramolecular chemistry, Chirality, Mechanical properties

## Abstract

In order to widen the use of soft materials in tissue engineering and life sciences, hydrogels with improved mechanical properties and controlled chirality are critical to achieve. A methodology is presented to enhance the mechanical properties and gain the control of chirality of two component hydrogels by merely varying the solution pH. pH change has been used as a way to ionize the specific functionalities into positive and negative charges. These positive and negative charges are crucial to provide a surge of electrostatic interactions to the components, imparting the improvement in stability and regulating their optical activity. Our goal is to throw light on the significance of opposite charges in the hydrogels for achievement of desired properties.

• Role of ionisable groups is crucial to control viscoelastic and optical properties of supramolecular hydrogels.
• Increasing the pH of the solution increases the number of negative ions by affecting the ionisable moieties, which interact with the positive charges in the solution.
• Zeta potential of both materials has been analysed to ensure the presence of charged species.

## Graphical abstract

**Figure:**
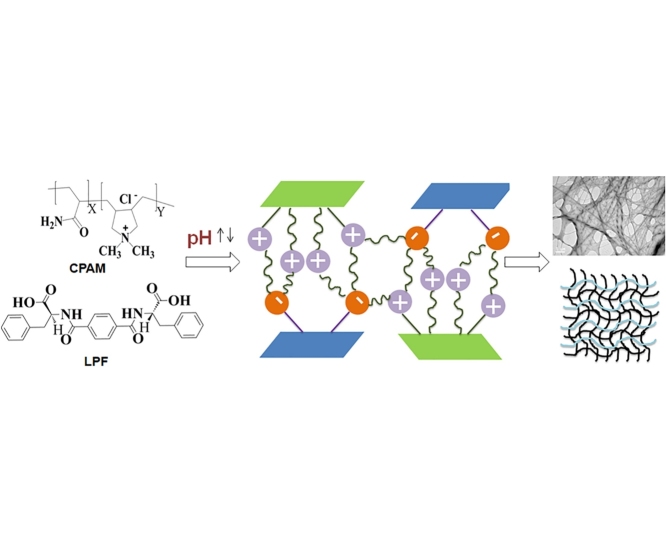

**Specifications Table**

**Table:** 

**Subject Area:**	Materials Science Chemistry Chemical Engineering
**More specific subject area:**	Supramolecular chemistry and nanoscience
**Method name:**	Method to improve mechanical properties and control chirality of supramolecular hydrogels
**Name and reference of original method:**	Y. Xie, X. Wang, R. Huang, W. Qi, Y. Wang, R. Su, et al., Electrostatic and aromatic interaction-directed supramolecular self-assembly of a designed Fmoc-tripeptide into helical nanoribbons, Langmuir 31 (2015) 2885-2894.

## Overview

Supramolecular hydrogels provide a fundamentally new strategy for material scientists to gain the control of soft materials’ properties by molecular engineering of wide set of substrates for their applications in diverse fields [1]. However, due to the presence of large water content, the stability and viscoelastic properties of supramolecular hydrogels are low which limits their applications in different fields such as in tissue engineering and cell culture because of direct influence of mechanical properties on tissue and cellular compatibility [2]. Along with the mechanical properties, controlled chirality of the supramolecular hydrogels is also crucial and useful in medical and biological fields, chiral recognition, chiroptical switches, optoelectronics and asymmetric catalysis [[3], [4], [5], [6], [7]]. Several methods including incorporation of inorganic nanomaterials and nanoparticles, polysaccharides, exfoliated graphene, functionalized dextran etc., has been used to improve mechanical properties of supramolecular hydrogels [[8], [9], [10], [11]]. But the issues posed by these methods such as enhanced viscosity, compatibility problems and difficult synthesis and homogenizing methods cannot be overlooked. To achieve controlled chirality different external stimuli such as light, temperature, solvent effects, metal ions, and sonication have been used. However, the requirement of presence of complex functionalities and coordination interactions by these methods can be difficult to meet.

**Scheme 1:**
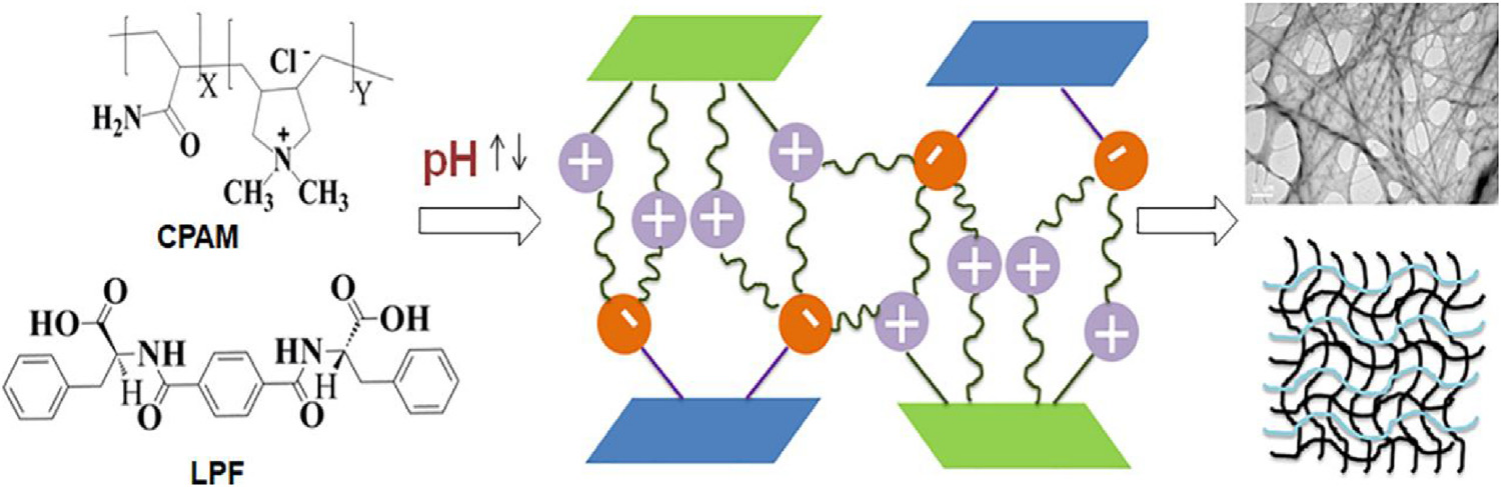
Schematic showing the effect of charges on properties of hydrogels.

A fast and efficient strategy to control the stability and chirality of the supramolecular hydrogels is through regulating the non-covalent interactions by varying the pH of the system. Incorporation of ionizable groups in the systems provides the opportunity to regulate the non-covalent interactive forces effectively [12]. The electrostatic interactions among the molecules influence hydrogen bonding and π-π interactions and enhance the stability and alter the chirality of the system (Scheme 1).

**Fig. 1:**
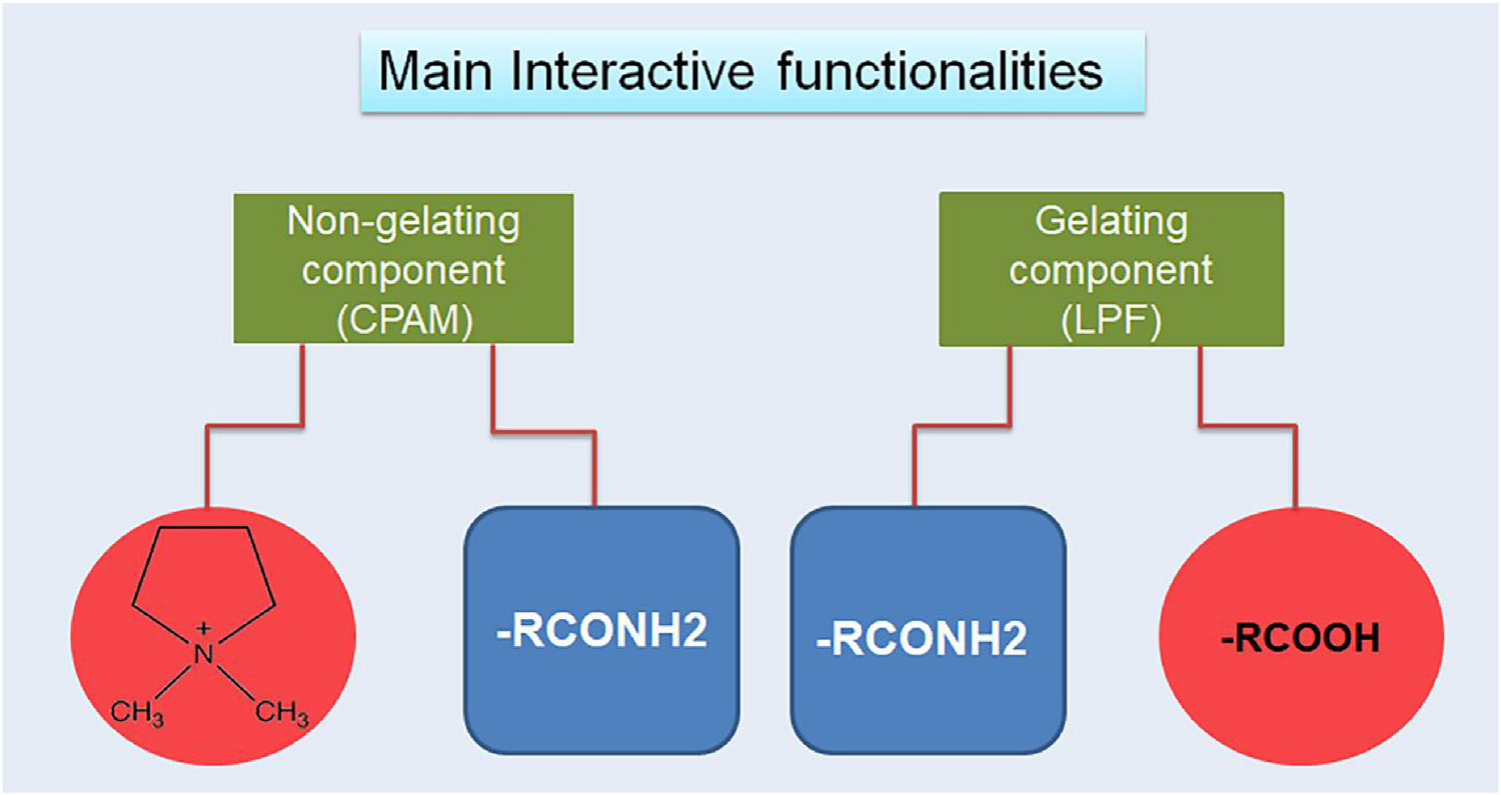
The main functionalities responsible for enhanced interactions upon changing the pH.

To test the hypothesis given above, two components have been used to prepare hydrogels. One component contains two carboxylic acid groups and is able to gelate water molecules i.e., LPF(C_26_H_24_N_2_O_6_) and other component is the polymeric component containing quaternary ammonium ions and amide groups i.e., CPAM (Poly(acrylamide-co-diallyldimethyl ammonium chloride) which cannot form gel alone (Fig. 1). Increase in the pH increases the negative ions by ionizing carboxylic acid groups which are capable to interact with the positively charged quaternary ammonium ions. The number of positive charges does not change considerably with the pH change due to the obvious presence of positive charges in the system. The interactions between charged components impart significant effect on stability and optical properties of hydrogels.

## Selection of appropriate concentration of non-gelating component

The concentration of gelating component has been fixed as 1 mol/L as compared to CPAM on the basis of critical gelation concentration. Different concentrations of CPAM have been used to test the gelation ability and synergistic assembly of both components. Solution of CPAM has been prepared at different molar concentrations ranging from 2 to 15 mol/L.

In this regard, we selected 4 mol/L CPAM solution to conduct the further experiments due to following reasons:

• The gelating component i.e., LPF dissolves in the CPAM solution with less efforts.
• 4 mol of the CPAM would give four quaternary ammonium ions in the solution which would interact with two carboxylate ions given by LPF forming a network of non-covalent interactions.

### Preparation of CPAM solutions at different pH

Commercially bought 20% wt. CPAM solution was used in the experiment. First the solution was diluted up to the concentration of 4 mol/L with the help of measuring cylinder. 1 M NaOH and HCl solution was used to adjust the pH of CPAM solution. Solutions at different pH ranging from 2 to 10 were prepared. pH meter has been used to check the solution pH.

• The pH of the solution starts to lessen after two to three days due to the interaction of atmospheric carbon dioxide. Try to check the pH every time before doing the experiment.

## Hydrogel preparation

### Equipment

5 mL vial

Heating gun

Sonicator

Vortex

First take 2 mg of LPF (1 mol LPF: 4 mol CPAM) in the glass vial and slowly add the CPAM solution of specific concentration. The gelating material is very hydrophobic, so it will be tough to dissolve it by heating alone. For that reason, use of vortex to mix the two components and sonicator to dissolve the LPF powder into the polymeric solution is recommended. One minute for vortex and 3–4 minutes of sonication to make the solution homogeneous is recommended. After sonication, take the vial in the wooden clippers and take it near heating gun. Pull down the glass window of the reaction chamber in order to prevent any damage because the vial may burst when subjected to heating for longer duration. Start to heat the vial along with gentle shaking. Avoid shaking too much; it may cause the gelating component to stick to the walls of the vial, resulting in no gelation. When the white powder gets dissolved in the solution and clear solution is appeared, stop heating and place the vial on room temperature for 20 min. After the specified time, hydrogel formation can be visualized by inverting the vial.

• Preparing the hydrogels at different pH values require different conditions. The hydrogels formation at acidic pH values is very tough. It is recommended to increase sonication time up to ten minutes, which will make the dissolution easier on heating and getting the clear solution will be less tiring.
• At pH 11, the hydrogel does not form and a clear solution remains behind due to too many OH^−^ groups which disturb the self assembly of gelator.

**Fig. 2:**
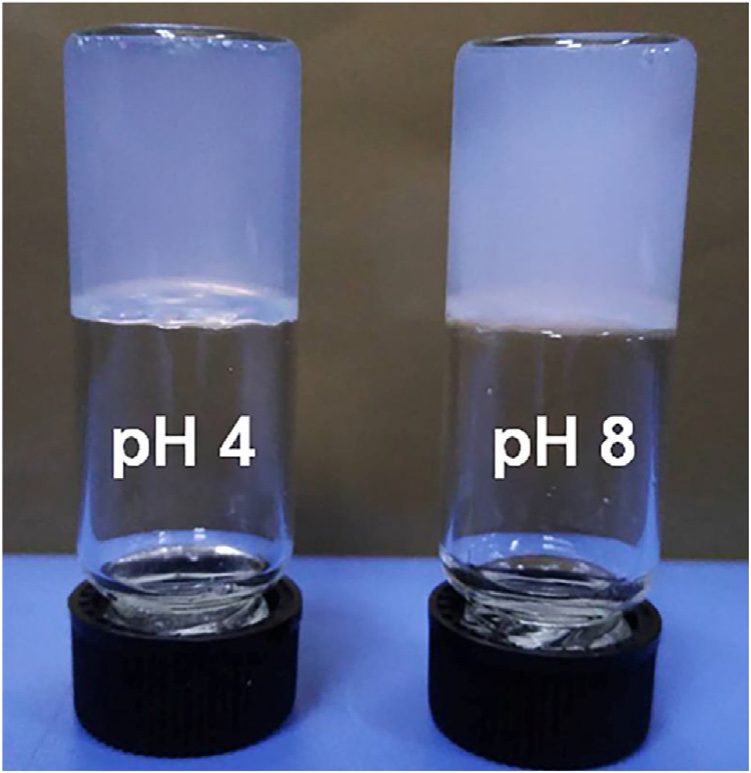
The change in appearance of hydrogels in acidic and basic environments.

The appearance of the hydrogel shows some changes with the increase in the pH (Fig. 2). The translucent appearance of the hydrogel in acidic environment turns to more whitish at higher pH values, pointing towards the changes in the stability.

## Method validation

**Fig. 3:**
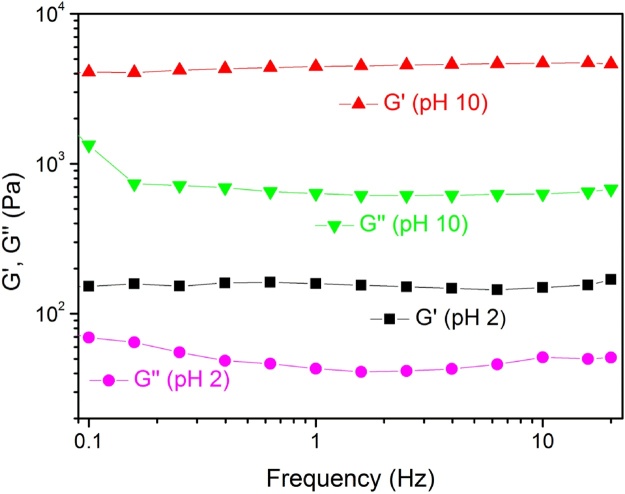
Frequency sweep of LPF-CPAM at pH 2 and pH 10. Frequency range used for conducting frequency sweep is.0.1–20 Hz.

Rheological measurements were carried to analyse the influence of pH on co-assembled hydrogels while CD measurements were analysed to check changes in the optical properties. Fig. 3 shows the change in loss and elastic modulus with increase in the pH. At pH 2, the mechanical properties of the hydrogels were very weak, showing elastic modulus of 154 Pa which is near to elastic modulus of LPF hydrogel found to be 92 Pa. The reason behind lower mechanical properties of LPF-CPAM at pH 2 is because too many protons (harsh conditions) in the solution affect the self assembly of the hydrogels [13], making them weaker in strength. When the pH reaches to 4, the value of elastic modulus shows considerable enhancement and reaches to 1433 Pa. This is due to the hydrogen bonding interactions among amide groups and carboxylic acid groups of both components. Moreover, at pH 7, the elastic modulus shows the value of 1839 Pa and reaches to 4087 Pa at pH 10 (Table 1, Fig. 3). The value of zeta potential was measured in order to account for the involvement of electrostatic interactions in the stability of co-assembled hydrogels (Table 1). It is evident from the data obtained (Table 1) that with increase in the pH, carboxylic acid groups in LPF undergoes ionization. The carboxylate ions formed interact with the positive ions present in the solution of CPAM and cause the increase in mechanical properties.

**Table 1:** Zeta potential and elastic modulus values obtained in different pH environments.

pH	Zeta potential of LPF (Mv)	Zeta Potential of CPAM (Mv)	Value of elastic modulus (Pa)
7	−28.	23.2	1839
8	−33.6	22	2802
9	−36	23	3313
10	−43.9	18	4087

**Fig. 4:**
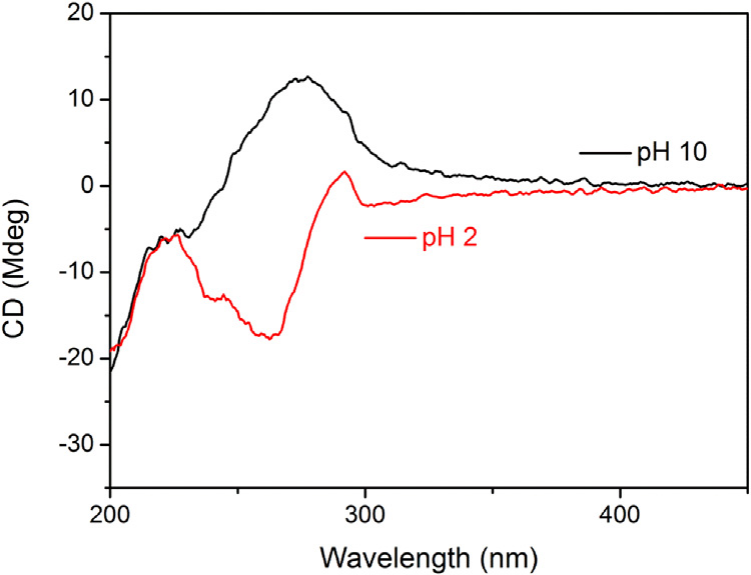
CD spectra of LPF-CPAM at pH 2 and 10 showing opposite CD signals.

Effect of pH on the chiral properties of co-assembled hydrogels was checked through analysis of CD spectra. It was found that at pH 2, positive CD signal was observed for LPF-CPAM. However, at pH 10, the CD signals goes into positive region with slight red shift. This is indicative of the different arrangement patterns at acidic and basic pH. The considerable changes in the CD spectra point towards effect of pH on co-assembly of both components, leading to enhanced electrostatic interactions which affect the packing behavior of hydrogels (Fig. 4).

So, it is concluded that pH change can be used as an effective way to control the chiral and mechanical properties which may widen the applications of soft materials in biology, medicine and tissue engineering.

### Failed methods

• Simple mixing of two components using pH as stimulus for gelation was also tried. First gelator solution was dissolved in 5 μL of 1 M NaOH solution in 5 mL glass vial and acidic CPAM solution was added into it. The solution was gently shaken in order to mix the two components and kept it still for 15 min to allow the gelation process. However, very weak hydrogel, possessing sticky texture was obtained which did not pass the vial inversion test. This may be due to the number of hydroxyl ions exceeding the required number limit.
• Effect of change of concentration of non-gelating component was also tested to improve the mechanical properties by expecting the increased hydrogen bonding between amide groups of non-gelating component and carboxylic acid groups of gelating components. However, with the increase in the concentration, considerable enhancement of the properties could not be achieved. The hydrogels did not form when the molar concentration was more than 15 mol/L. By using concentration ranging from 2 to 15 mol/L, the hydrogel formation occurred but there was no considerable difference in their properties which was analyzed using rheological measurements and CD spectroscopy.
